# Mast cell-mediated immune regulation in health and disease

**DOI:** 10.3389/fmed.2023.1213320

**Published:** 2023-08-17

**Authors:** Kottarappat N. Dileepan, Vineesh V. Raveendran, Rishi Sharma, Harita Abraham, Rajat Barua, Vikas Singh, Ram Sharma, Mukut Sharma

**Affiliations:** ^1^Division of Allergy, Clinical Immunology and Rheumatology, Department of Medicine, The University of Kansas Medical Center, Kansas City, KS, United States; ^2^Department of Medicine, School of Medicine, University of Missouri, Kansas City, MO, United States; ^3^Cardiology Section, Kansas City Veterans Affairs Medical Center, Kansas City, MO, United States; ^4^Neurology Section, Kansas City Veterans Affairs Medical Center, Kansas City, MO, United States; ^5^Research and Development Service, Kansas City Veterans Affairs Medical Center, Kansas City, MO, United States; ^6^Midwest Veterans’ Biomedical Research Foundation (MVBRF), Kansas City VA Medical Center, Kansas, MO, United States

**Keywords:** cardiovascular disease, neuroinflammation, cigarette smoking, bacterial infection, SARS-CoV-2 disease, atherosclerosis, allergic disease, skin and wound-healing

## Abstract

Mast cells are important components of the immune system, and they perform pro-inflammatory as well as anti-inflammatory roles in the complex process of immune regulation in health and disease. Because of their strategic perivascular localization, sensitivity and adaptability to the microenvironment, and ability to release a variety of preformed and newly synthesized effector molecules, mast cells perform unique functions in almost all organs. Additionally, Mast cells express a wide range of surface and cytoplasmic receptors which enable them to respond to a variety of cytokines, chemicals, and pathogens. The mast cell’s role as a cellular interface between external and internal environments as well as between vasculature and tissues is critical for protection and repair. Mast cell interactions with different immune and nonimmune cells through secreted inflammatory mediators may also turn in favor of disease promoting agents. First and forefront, mast cells are well recognized for their multifaceted functions in allergic diseases. Reciprocal communication between mast cells and endothelial cells in the presence of bacterial toxins in chronic/sub-clinical infections induce persistent vascular inflammation. We have shown that mast cell proteases and histamine induce endothelial inflammatory responses that are synergistically amplified by bacterial toxins. Mast cells have been shown to exacerbate vascular changes in normal states as well as in chronic or subclinical infections, particularly among cigarette smokers. Furthermore, a potential role of mast cells in SARS-CoV-2-induced dysfunction of the capillary-alveolar interface adds to the growing understanding of mast cells in viral infections. The interaction between mast cells and microglial cells in the brain further highlights their significance in neuroinflammation. This review highlights the significant role of mast cells as the interface that acts as sensor and early responder through interactions with cells in systemic organs and the nervous system.

## 1. Introduction

The immune system consists of macrophages, dendritic cells, monocytes, natural killer cells, basophils, eosinophils, neutrophils, T and B lymphocytes and mast cells, and a group of proteins that constitute the complement system (1). Among these cells, mast cells draw special attention due to their presence at strategic locations, expression of several stimulatory as well as inhibitory receptors, and their physiological and pathobiological functions based on the microenvironment. Mast cells are tissue-resident guard cells localized at strategic barrier locations, unlike monocytes, neutrophils, and T and B lymphocytes that circulate in the blood (2). Paul Ehrlich, who discovered mast cells, was also the first to describe their presence in the perivascular areas (3). Mast cells in rodents are grouped under two categories, namely the large connective tissue mast cells (CTMCs) and the mucosal mast cells (MMCs) although other organ specific types have been identified. Human mast cells (MCs) are also classified into two groups based on the type of protease(s), namely tryptase and chymase-positive (MC_tc_) and tryptase-only-positive mast cells (MC_t_). Classification of mast cells continues to be refined with advancing computing power for analysis (4).

Mast cells originate from hematopoietic progenitor cells which express c-kit (CD117) (5), the receptor for stem cells factor (SCF) and CD34 but lack FcεR1 (6–9). Circulating mast cell precursors migrate to different tissues through adhesion contacts to a network formed by integrins (10–12). Depending on the microenvironment, mast cells acquire specific phenotypic characteristics and expression of receptors. For example, FCγRIIb is expressed by mast cells of gastrointestinal tract but not by mast cells of skin (13). Mature mast cells are present in the areolar connective tissue space of many organs, such as the adventitial layer of blood vessels, skin, nerve fibers, smooth muscles, airways, gastrointestinal tract, and adipose tissue (14–16).

A characteristic feature of mast cells and other granulocytes (neutrophils, basophils, and eosinophils) is the regulated degranulation of cytoplasmic granular bodies (granule exocytosis) and their ability to regranulate (17). Mast cell degranulation is now a subject of intense investigation because of its therapeutic potential (18). Mast cells can also utilize the classical/constitutive secretory pathway to release mediators that modulate the innate and adaptive immune systems (19). Mast cells can also release DNA to form extracellular traps (MCET) in response to certain microorganisms (20, 21). MCET formed by mast cells have been shown to contain histones, tryptase and LL-37 (20, 22, 23). These characteristics enable mast cells to function as frontline responders to the toxins released by the invading viral, bacterial, or fungal pathogens, insects, and parasites. It is noteworthy that mast cell proteases are capable of degrading insect and snake venom toxins by releasing carboxypeptidase A (CPA3) and thus increasing resistance to their toxic effects (24). Mast cells may also act in synergy with exogenous toxins to amplify inflammatory responses. However, aging and senescence may significantly affect mast cell number and function (25).

In this article, we highlight mast cell receptors and mast cell-generated mediators that regulate inflammatory responses. These mediators enable mast cells to act as pro-and anti-inflammatory effectors to promote mobilization and proliferation of other immune cells. Next, using their perivascular localization as a point-of-reference, we address the role of mast cells under physiological conditions as well as in representative diseases where mast cells orchestrate cross-talks between different cell types and manage immune homeostasis. We discuss the role of mast cells in early vascular inflammation with and without co-morbidities (chronic/sub-clinical infections) and long-term progression to atherosclerosis. Lifestyle choices affect both the severity and outcome of vascular disease with or without infection. Using cigarette smoking as a lifestyle choice model, we outline recent research connecting cigarette smoking with mast cells, vascular inflammation, and bacterial toxins. Role of mast cells in virus-induced pathologies is an emerging field. Here, we discuss the evidence that links mast cells to changes in the capillary and alveolar structure–function following SARS-CoV-2 infection. Since mast cells are uniquely positioned to connect the central nervous system with changes in peripheral organs through the vasculature, we summarize growing evidence that demonstrates interaction between mast cells and brain (microglial) cells that influences neuro-inflammation.

## 2. Main classes of mediators released by mast cells

### 2.1. Overview of the mechanisms for release of mediators

**Figure 1:**
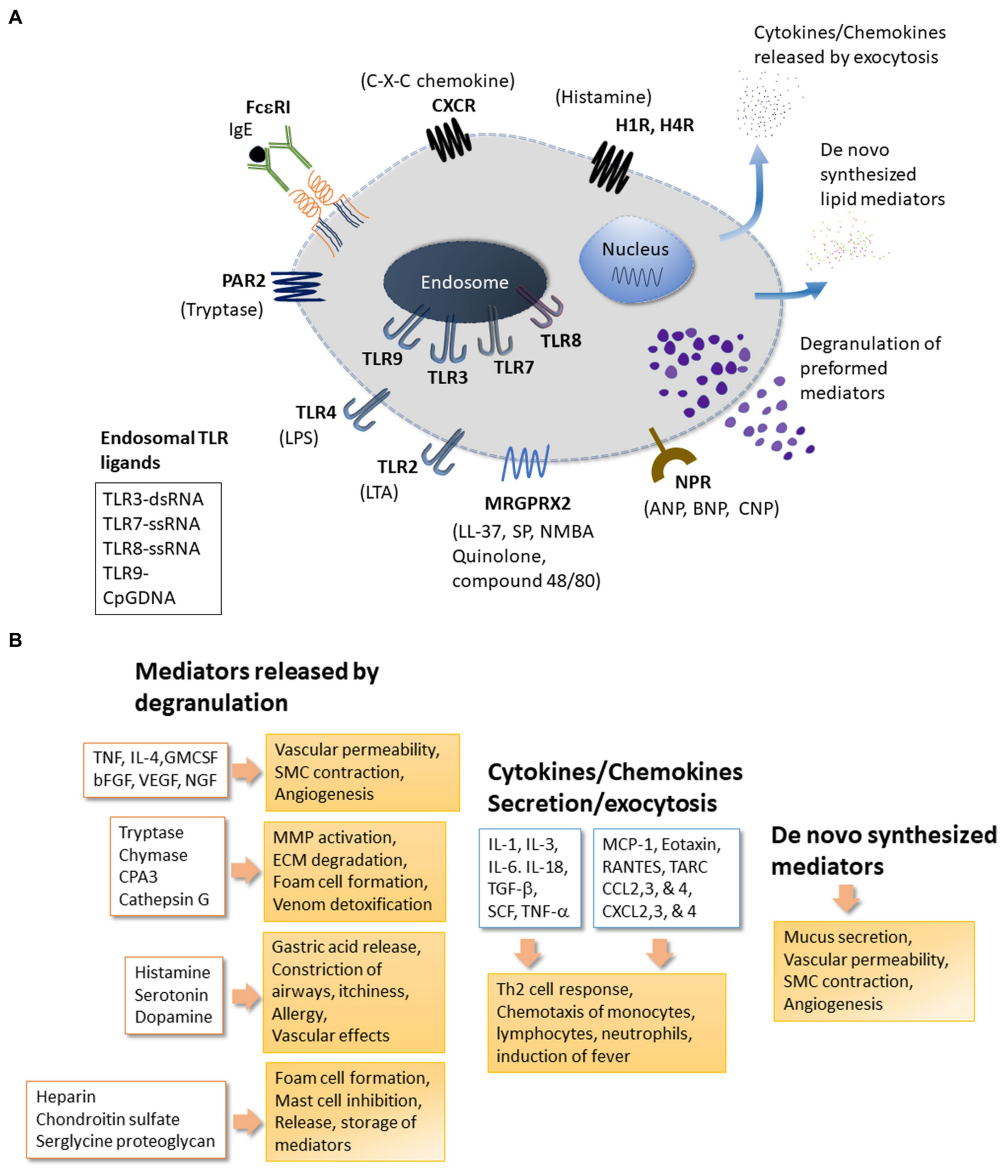
Mast cell receptors and products. Panel **(A)** depicts some of the major receptors expressed on mast cells. Respective ligands of these receptors are also shown. The receptors shown here include the high affinity FceRI for IgE; MRGPRX2: Mas-related G-protein coupled receptor-X2 that binds quinolones and compound 48/80 among other ligands; TLRs (Toll-like receptors) at the plasma membrane (TLR2 and TLR4) and at endosomes (TLR3 and TLR7-9) that bind to various exogenous and endogenous molecules in a pattern recognizing manner; NPR for neuropeptide ligands ANP, BNP or CNP; Protease Activated Receptors PARs; Histamine Receptors HRs for histamine; CXC-Chemokine Receptor CXCR. Other important receptors expressed on mast cells (not shown here) include receptors for Kit (stem cell factor receptor), Complement (C3a and C5a); alarmins IL-33 (ST2, IL-1 family receptor) and Epithelial cell derived Thymic stromal lymphopoietin (TSLP); the platelet-activating factor (PAF); vascular endothelial growth factor (VEGF); nerve growth factor (NGF); fibroblast growth factor (FGF); Interleukins (IL); Transforming growth factor beta (TGF-b); Thymus and activation-regulated chemokine (TARC); Prostaglandins (PGs); Cysteinyl leukotrienes (CysLT). Mast cell regulator/inhibitory receptors containing immunoregulatory tyrosine inhibition motifs (ITIMs) including IgE receptor Fc-gamma-RIIb, CD300a, CD200 R1, platelet-endothelial cell adhesion molecule 1 (PECAM-1), paired immunoglobulin-like receptor B (PIR-B), the c-lectin mast cell function-associated antigen (MAFA), sialic acid-binding immunoglobulin-like lectins (Siglecs), and leukocyte immunoglobulin-like receptor 4, subfamily B, member 4 (LILRB4). Panel **(B)** mast cells release many effector molecules through different mechanisms. Degranulation is a robust mechanism for mast cells to release pre-formed molecules of several chemical classes. However, release of mediators through degranulation is a complex process that may vary with regard to the composition of granules or the duration of release resulting in a fine control of mast cell response to their surroundings. Release of molecules including cytokines and chemokines may involve the classical secretory pathway or other mechanism for exocytosis. Molecules such as prostaglandins and leukotrienes are synthesized *de novo* in response to stimuli. Mediators released by mast cells enable mast cells to respond to many physiological and pathological conditions. bFGF, Basic Fibroblast Growth Factor; CCL2,3, and 4, Chemokine (C-C motif) Ligand 2, 3 and 4; CPA3, Carboxypeptidase-3; CXCL2,3 and 4-Chemokine (C-X-C motif) Ligand 2, 3 and 4; cysLTs, Cystinyl Leukotrienes; GM-CSF, Granulocyte Macrophage Colony-Stimulating Factor; IL-1, IL-3, IL-4, IL-6, IL-18, Interleukins 1, 3, 4, 6, 18; NGF, Nerve Growth Factor; MCP-1, Monocyte chemoattractant protein-1 (CCL2); PAF, Platelet Activating Factor; PGs, Prostaglandins; RANTES, Regulated upon Activation, Normal T cell Expressed and Secreted aka (CCL5); SCF, Stem Cell Factor; SMC, Smooth Muscle cells; TARC, Thymus and Activation Regulated Chemokine; TGF-β, Transforming Growth factor-β; TNF, Tumor Necrosis Factor; VEGF, Vascular Endothelial Growth Factor/vascular permeability factor.

Mast cells package pre-formed mediators in granules that are released upon activation. Mast cells also secrete several mediators through the secretory pathway leading to exocytosis of such molecules as large vesicles, microvesicles or exosomes (26, 27). Besides activation-induced secretion of mediators, human skin mast cells can constitutively and spontaneously secrete pro-angiogenic factors (28). Figure 1 provides a snapshot of pre-formed, cytokines/chemokines and *de novo* synthesized mediators released as well as some key receptors expressed by mast cells. Mediators stored in the granules are diverse chemical entities grouped as biogenic amines (histamine, serotonin, dopamine) (29–32), proteases (serine proteases, aspartic acid proteases, cysteine proteases, metalloproteinases) (33, 34), peptidoglycans (heparin, chondroitin sulfate) (35), many cytokines (TNF, IL-4 etc.) (36, 37), and growth factors (GM-CSF, bFGF, VEGF, NGF) (38). Newly synthesized inflammatory mediators include lipid mediators (LTB4, LTC4, PAF, PGD2), neuropeptides (CRH, VIP) (39), growth factors (PDGF, GnRH) (38), chemokines (MCP-1, eotaxin, TARC, RANTES) (40, 41), and cytokines (IL-1, IL-3, IL-6, IL-18, SCF, TGF-β) (27, 36, 37). Table 1 provides a list of mediators released by mast cells, their receptors, and their significance in disease. Such wide array of inflammatory mediators and the ability to release them through diverse mechanisms enable mast cells to perform a variety of functions including regulation of innate and adaptive immune mechanisms, participation in host defense against invading pathogens, parasites, venom detoxification and elimination of cancer cells (2, 172–178).

**Table 1:** Mast cell-released mediators act through their receptors on target cells in various tissues and contribute to physiological functions as well as pathophysiological changes.

Mast cell mediators and their receptors on target cells	Physiological functions		Associated diseases
Histamine/H1R H2R H3R H4R	Blood vessels and blood cells	Vasodilation, increased permeability (42, 43), modulation of functions of monocytes/ macrophages (44), eosinophils (44), T-cells (45), neutrophils (46), endothelial cells (47–50)	Allergies (51), asthma (52) eczema (53), urticaria (hives) (54), anaphylaxis (55), mastocytosis (56), Cardiovascular diseases (57–60), coronary artery disease (61)
	Smooth muscle cells	Bronchoconstriction (62)	Asthma (62), allergies (63, 64),
	Gastrointestinal tract	Increased secretion, increased motility, and contraction (65)	Inflammatory Bowel Disease (IBD), Irritable Bowel Syndrome (IBS) (66, 67)
	Skin	Pruritus (itching), redness, swelling (68)	Eczema, urticaria (53, 54)
	Nerves	Stimulation, increased pain sensitivity (68, 69)	Migraines (70, 71), fibromyalgia (72)
Cytokines
IL-1/IL-1R	Induces fever, activates T and B lymphocytes (73), promotes vascular permeability, induces the expression of other cytokines (74–76)		Arthritis (77), Covid 19 (74–76)
IL-2/IL-2R	Stimulates T cell proliferation and differentiation (78)		Allergic dermatitis, chronic inflammation (79)
IL-4/IL-4R	Stimulates IgE production, enhances Th2 response (80), fibroblast activation (81)		Allergic reactions, asthma, eczema, fibrosis (80)
IL-5/IL-5R	Stimulates eosinophil activation, maturation, and migration (82)		Asthma, eosinophilic disorders (82)
IL-6 IL-6R	Differentiation of T-and B-lymphocytes, and proliferation of smooth muscle cells (83), monocyte to macrophage maturation (84), induces acute phase response (85)		Atherosclerosis (86, 87), cancer (88)
IL-8/IL-8RA/B	Chemotaxis of neutrophils and lymphocytes (89), cell adhesion (90), activation of neutrophils (91)		Atherosclerosis (92), cancer (93, 94)
IL-13 IL-13R	Stimulates mucus production (95), enhances Th1 response (96)		Asthma, dermatitis (96)
IL-31 IL-31R	Proliferation of epidermal cells (97)		Atopic dermatitis (98), osteoporosis
MCP-1	Chemotaxis and activation of monocytes (99)		Atherosclerosis (100)
TNF/TNFR1 TNFR2	Activates macrophages, increases vascular permeability of endothelial cells (101, 102), and induces glomerular capillary albumin permeability (103), activates T cells (95)		Rheumatoid arthritis (RA) (104), psoriasis (105), cancer (88)
TGF-β/TGF-βR	Inhibits inflammatory response, promotes wound healing, Treg differentiation (106, 107)		Autoimmune diseases (108), cancer (88)
Prostaglandins
PGD2/PTGDR1(DP1) PTGDR2(DP2)	Vasodilation, increased vascular permeability, pain sensitization (51, 109)		Allergies, asthma, eczema, rhinitis, urticaria (51)
PGE2/PTGER1(EP1) PTGER2(EP2)	Vasodilation (110), reduces inflammation, stimulates Th2 response (111)		Allergic asthma (112)
PGF2α/PTGFR(FP)	Contraction of smooth muscle (113)		Rheumatoid arthritis (114)
PGI2/PTGIR(IP)	Vasodilation (115), inhibits platelet aggregation, inhibits smooth muscle contraction (116)		Pulmonary hypertension (116), Raynaud’s phenomenon (117), scleroderma (117)
TxA2/Thromboxane receptor(TP)	Vasoconstriction (118), promotes platelet aggregation (119)		Cardiovascular disease (120), asthma (121)
Leukotrienes
Cys-LTs-LTC4, LTD4, and LTE4/cysTL1 and cysLT2	Bronchoconstriction (122), increased vascular permeability, mucus secretion, chemotaxis (123)		Asthma (123), chronic obstructive pulmonary disease (COPD) (124), rhinitis, eczema, urticaria, anaphylaxis, IBD (125)
LTB4/BLT1 and BLT2	Induces chemotaxis and activation of neutrophils (126) and eosinophils (127)		Psoriasis (128), RA (129), IBD (130)
5-HETE, 12-HETE, 15-HETE/Oxer, GPR31, BLT2	Induces chemotaxis and activation of neutrophils (131) and eosinophils (132)		Asthma (133)
Proteases
Chymase/PAR2	Blood vessels	Vasoconstriction (134), extracellular matrix degradation (135) Angiotensin II formation in heart (136)	Hypertension (137) atherosclerosis (138, 139), cardiovascular disease (140), fibrosis (141), COPD (142), cancer (143)
	Smooth muscle	Contraction (123), Extracellular matrix degradation (135)	Asthma (144), atherosclerosis (145)
	Gastrointestinal tract	Extracellular matrix degradation (135)	IBD (146)
	Skin	Extracellular matrix degradation (135)	Aging (147)
	Nerves	Stimulation (39)	Pain (148), itching, mastocytosis (149)
Tryptase/PAR2	Blood vessels	Vasodilation, increased permeability (150), angiogenesis (151)	Allergies, asthma, eczema, urticaria (Hives), atherosclerosis (139)
	Smooth muscle cells	Contraction (152), proliferation (153)	Asthma (154), allergies, mastocytosis (155)
	Gastrointestinal tract	Increased secretion, increased motility, contraction (156)	IBD, IBS, mastocytosis (157)
	Skin	Pruritus (itching), redness, swelling (158)	Eczema (159), urticaria (160)
	Nerves	Stimulation, increased pain sensitivity (161)	Migraines, fibromyalgia (162), IBS, mastocytosis (161)
Carboxypeptidase-A3	Degradation of venom peptides (163, 164)	reduces inflammatory response by destroying peptide signals (163)	Inflammatory diseases, asthma (163)
Heparin	Anticoagulant (165)	Inhibits coagulation cascade (165)	Deep vein thrombosis, pulmonary embolism (166)
	Anti-inflammatory (167)	Inhibits release of histamine and other inflammatory mediators (168)	Allergic reactions (168), asthma (169)
	Immunomodulatory (170)	Modulates immune response (170)	Heparin-induced thrombocytopenia (171)

Generally, the type of mediators released by mast cells are determined by the receptor [s] activated by specific ligands and the microenvironment. However, the composition, chronology of the release of granules, their cargo and multiple mechanisms for secreting mediators contribute to variations to this generalization (27). The pleiotropic nature of mast cells is also determined by the diversity of receptors, degree of ligand-specificity of receptors and receptor interactions and the effect of released mediators with the microenvironment (179). For example, IgE binds with high affinity to FcɛRI (tetrameric FcεRIαβγ_2_) expressed on mast cell, basophils, and gastrointestinal mucosa (180). However, IgE can also bind with lower affinity to FcεRII (or CD23, a C-type lectin) and to ε-binding protein εBP (or galectin 3) (181, 182). Such heterogeneity of the cells carrying different receptor isoforms for the same ligand creates varying response to IgE. Also, ligands that bind distinct receptor(s) may induce strong (allergy-type) responses without involving IgE. For example, quinolone containing tetrahydroisoquinoline (THIQ) motif (ciprofloxacin, vancomycin, morphine, rocuronium and others) bind to mast-cell specific Mas-related G protein-coupled receptor (MRGPRX2) and may cause severe allergy-type symptoms without involving IgE (183). In addition, mast cells may release mediators in a selective, piece-meal or discriminatory manner. Selective/differential release of mediators implies a preferential release of a cytokine or neurotransmitter without/before secretion of other mediators and/or without degranulation, e.g., serotonin release without histamine and without degranulation (74, 184, 185). The following sections outlines representative secretory products of the mast cell and their roles in inflammation with special emphasis on vascular inflammation.

### 2.2. Histamine

Histamine is a vasoactive amine synthesized and stored in the cytoplasmic granules of human basophils and mast cells. It is one of the major secretory products of mast cells that is well-recognized for its participation in allergic and hypersensitivity reactions. Histamine alone as well in combination with other mast cell products regulates vasodilation, bronchoconstriction (186, 187) and modulate the functions of monocytes/macrophages (188, 189), eosinophils (44, 188), T-cells (45), neutrophils (46), and endothelial cells (47–50).

Effects of histamine on physiological processes are mediated through a family of G-protein-coupled receptors, H1, H2, H3, and H4 (190). H1 receptors are highly expressed in many cell types including endothelial cells, smooth muscle cells, neuronal cells, respiratory epithelial cells, hepatic cells, dendritic cells, lymphocytes, and mast cells. Histamine contributes to vasodilation (through histamine-H1R-CycAdenosine axis), arteriolar constriction (through histamine-H1R-thromboxane axis) (118), angiogenesis, and vascular permeability. Histamine acting through the H1 receptor modulates inflammatory and hypersensitivity responses (47, 191–193). The H2 histamine receptor participates in the stimulation of gastric acid secretion in the gut and in the regulation of cytokine production by cells in cardiac, smooth muscle, and immune system (189, 194–197). The H2 receptor is expressed in a wide array of cells including B cells, T cells, dendritic cells, gastric parietal cells, smooth muscle cells as well as in the brain and cardiac tissues (192). The H3 histamine receptor is expressed in histamine-containing neurons of the brain (198) and functions by coupling to G_αi/0_. The H4 receptor has approximately 40% homology to the H3 receptor and is highly expressed in bone marrow and leukocytes, and moderately expressed in spleen, thymus, lung, small intestine, colon, and heart (199–201). The H4 receptor is also expressed in the central nervous system and in certain cancer cells. The H4 receptor-signaling is associated with G_αi/0_. It is noteworthy that the physiological outcomes of histamine action engaging different subtypes of histamine receptors are distinct and cell-specific.

The significance of histamine and its receptors in cardiovascular disease (CVD) has been proposed in several pioneering studies (57, 58, 202). Our studies have shown that histamine acting through H1 receptor stimulates the expression of IL-6 and IL-8 (47) and COX2 (49) in human coronary artery endothelial cells (HCAEC) *in vitro* which suggests that it can act as an important vascular inflammatory signal. Interestingly, the effects of histamine on these parameters were synergistically enhanced by lipopolysaccharide (LPS), peptidoglycan (PGN) and lipoteichoic acid (LTA) (47, 48). Our studies also demonstrated that LPS would enhance H1 receptor expression in endothelial cells (50). In addition, histamine can induce smooth muscle cell migration and proliferation (203, 204), and plays a role in intima thickening in a mouse model (205).

A direct relationship between histamine and vascular inflammation is evident from the observation that coronary arteries of patients with ischemic heart disease contain more mast cells and histamine than normal vessels (57). The role of histamine in vascular disease is further supported by the presence of elevated levels of histamine in the coronary circulation of patients with variant angina (206). Takagishi et al. have further shown increased expression of H1 receptor mRNA in smooth muscle cells of intima/media in the atheroma. Many recent reviews have focused on the role of histamine in coronary arterial disease (CAD) (61, 207–210).

### 2.3. Mast cell proteases

On a weight and molar basis, enzymatically active neutral proteases are the major protein constituents released from activated mast cells (175). Tryptase, chymase, and carboxypeptidase A, in various combinations, represent the three major proteases in the granules of mast cells (211). Expression of tryptases and chymases is highly specific for mast cells but proteases including cathepsins G, C, and L are present in various cells of inflammatory process. Both mast cells and basophils express carboxypeptidase A3 but proteases like mastin are mostly restricted to basophils (33). As mentioned earlier, the unique pattern of protease expression is the basis to classify heterogeneous mast cell populations broadly into MC_TC_ and MC_T_ (212–216). The role of mast cell chymase and tryptase in the progression of atherosclerosis has been extensively studied by Kovanen and colleagues (217–220) and by Bot and collaborators (221, 222).

Human tryptases are heterogeneous proteins expressed by alleles of α, β, and γ genes (223). Human mast cell tryptase can induce IL-8 and intercellular adhesion molecule 1 (ICAM-1) expression in bronchial epithelial cells (224), induce IL-8 production in endothelial cells (225), stimulate collagen synthesis and chemotaxis in fibroblasts (226), and initiate angiogenesis (227). Mast cells have been shown to modulate cardiomyocyte contractibility via release of tryptase, which activates protease-activated receptor 2 (PAR2) (228, 229). Tryptase by activating PAR2 also plays a prominent role in LPS-induced neutrophil recruitment and lung inflammation in a mouse model (230). Furthermore, increased numbers of mast cells and extracellular tryptase within the atherosclerotic plaques during initial stages of calcification have been demonstrated (231). Presence of mast cells in the human heart (232) and the recognition of mast cell chymase as a major pathway for the generation of angiotensin II (233, 234) support the role of mast cells in both myocardial and vascular functions. In addition, tryptase released from mast cells has been shown to activate sensory nerves to release substance P and mast cell chymase activates angiotensin-renin pathway (235). Mast cell chymase and carboxypeptidase A degrade low-density lipoprotein (236, 237). Recombinant mouse mast cell protease-6 independently induces infiltration of neutrophils *in vivo* and stimulates IL-8 production by cultured endothelial cells (238). These findings underscore the importance of mast cell-derived serine proteases in vascular inflammation and atherogenesis.

Our studies show that mast cell proteases and endotoxin synergistically activate human endothelial cells to generate IL-6 (239), and IL-8 (240). Chemokines and IL-6 play significant roles in the recruitment of inflammatory cells to the vessel wall. Mast cell activation leads to increased recruitment of leukocytes to the plaque with neutrophils as the predominant inflammatory cells in response to IL-8 (241). In addition, mast cell activation enhances the expression of adhesion molecules by endothelial cells which sets the stage for further increase in the transmigration of leukocytes to the plaque (242). Thus, endothelial activation induced by bacterial toxins and the subsequent overproduction of cytokines and chemokines can promote vascular inflammation independently as well as synergistically with mast cell proteases.

### 2.4. Heparin

Heparin is another component of mast cell granules with a wide range of biological functions including modulation of the release of mediators (243–245). In addition to its interaction with antithrombin III, heparin interacts with protease factor XII of the coagulation cascade. This interaction results in the production of bradykinin causing blood vessel dilatation, adhesion of various cell types to vascular endothelium, and increased vascular permeability leading to edema (246, 247). Heparin is also vital for the mast cell-mediated angiogenesis (248–250), and for vascular inflammation by binding to molecules such as polycationic peptides and chemokines (251, 252).

### 2.5. Major cytokines and chemokines

TNF is most abundant among the cytokines released by activated mast cells (101, 253). TNF causes immediate activation of macrophages, modulates the effects of other cytokines, increases vascular permeability of endothelial cells (101, 102), and causes increases in glomerular capillary albumin permeability *in vitro* (103). It is well-established that TNF facilitates vascular inflammation by increasing the expression of adhesion molecules resulting in increased binding of leukocytes and other immune cells to endothelial cells (254). In combination with IFNγ, TNF can increase vascular permeability by disrupting the cell-junction proteins (255). In addition, activated mast cells release other *de novo* synthesized cytokines including IL-1, IL-6, IL-8, and MCP-1 (256, 257).

Interleukin-6 (IL-6) is a pleiotropic cytokine secreted by a variety of cells including mast cells and endothelial cells and is known to stimulate the proliferation and differentiation of T-and B-lymphocytes, and smooth muscle cells (83). IL-6 has been shown to contribute to allergic conditions. *In situ*-derived human skin mast cells express functional membrane-bound IL-6 receptors. IL-6 enhances FcεRI-induced COX-2 expression and potentiates PGD2 biosynthesis through a STAT-3 dependent mechanism (258). In support of a pro-angiogenic role for mast cells, IL-6 was found to induce the expression of VEGF and MCP-1 in *in situ*-derived human skin mast cells (28, 258). It also initiates an acute phase response and is implicated in many inflammatory and autoimmune diseases (259). Elevated levels of IL-6 gene transcripts were found in atherosclerotic lesions of genetically hyperlipidemic rabbits (260). Atherosclerotic human arteries express 10 to 40-fold higher IL-6 mRNA than non-atherosclerotic arteries, and the thickened intimal layers of atherosclerotic vessels have higher number of IL-6 gene transcripts (86). Recently, IL-6 was shown to increase mast cell proliferation by down-regulation of suppressor of cytokine signaling 3 (SOCS3) and suppression of the hydrolysis of soluble IL-6 receptor resulting in a more reactive mast cell phenotype (261). Attenuation of IL-6 secretion has been proposed as a therapeutic approach for mast cell-related diseases such as mastocytosis (261).

Mast cells also release IL-8, a chemokine with a pivotal role in chemotaxis of neutrophils and inflammation. IL-8 was shown to be spontaneously secreted by human skin-derived mast cells (28). The diverse biological properties of IL-8 include chemotaxis of neutrophils and lymphocytes (89), regulation of cell adhesion (90), and activation of neutrophils (91). These pioneering studies implicate IL-8 as a key factor in the pathogenesis of vascular disease. In addition to its well-recognized chemoattractant properties, IL-8 is a potent angiogenic factor found at high concentrations in atherosclerotic lesions and macrophages collected from patients with the disease (92).

Monocyte chemoattractant protein-1 (MCP-1/CCL2) is a chemokine which is also released by mast cells. MCP-1 is a member of the CC chemokine family which attracts and activates monocytes (99). Mast cell degranulation induces production of MCP-1 and IL-8 in endothelial cells that is further amplified by tryptase (262). MCP-1 is important for allergen-specific activation of mast cell and acute phase inflammation and, it plays a critical role in the initiation and development of atherosclerotic lesions as it recruits monocytes into the sub-endothelial layers of the vessel wall (263, 264). Besides sub-endothelial macrophages and smooth muscle cells, endothelial cells are a major source of MCP-1 in atheromatous plaques (263, 264). Abundant amounts of MCP-1 have been detected in atherosclerotic lesions (265), and increased expression of MCP-1 mRNA has been detected in endothelial cells, macrophages, and vascular smooth muscle cells of human atherosclerotic arteries (86, 266). Studies using MCP-1-deficient and MCP-1 receptor-deficient mice have demonstrated decreased presence of macrophages and a reduction of atherosclerotic lesions (267).

Mast cells release pruritogenic cytokine IL-31 upon activation by antimicrobial peptides [e.g., human beta-defensins, cathelicidin, LL-37] (268, 269) and form extracellular traps by releasing DNA, like neutrophil extracellular traps, to inhibit bacterial growth (21). Overall, cytokines and chemokines released by mast cells enable rapid recruitment of neutrophils to the site of infection.

### 2.6. Major lipid mediators

Mast cells express both cyclooxygenase 1 and 2 (COX1 and COX2) (270) that catalyze the oxygenation of arachidonic acid to PGG_2_/PGH_2_ which are used by specific prostaglandin (PG) syntheses to generate PGE_2_, PGD_2_, PGF2α, PGI_2_, and TXA_2_. Among the PGs, PGI_2_, and TXA_2_ are well-recognized for their role in cardiovascular diseases (271–273). Prostacyclin (PGI_2_), is a potent vasodilator and an inhibitor of leukocyte adhesion, and platelet aggregation, and therefore, plays a protective role atherothrombosis (274). TXA_2_, in contrast, is a potent inducer of vasoconstriction, platelet activation and platelet adhesion (274). COX2 contributes significantly to systemic PGI_2_ synthesis in humans (275, 276), although COX1 also contributes to vascular PGI_2_ synthesis (272, 277). PGI_2_ has a protective role in vascular remodeling by smooth muscle cells and its absence leads to an increased intima/media ratio in response to vascular injury following disruption of the gene for the PGI_2_ receptor in mice (278). We have demonstrated that histamine induces the expression of COX2, but not COX1, in endothelial cells resulting in enhanced production of PGI_2_, indicating the vasodilatory effects of mast cells in vascular homeostasis (49). Since PGI_2_ and TXA_2_ act on vascular endothelium in opposing manners, their relative concentrations in the microenvironment and systemic circulation are critical for cardiovascular protection. These findings underscore the importance of mast cells in both cardiovascular health and cardiovascular disease.

Leukotrienes (LTs) belong to another group of arachidonic acid metabolites secreted by mast cells. LTs [LTA to LTE] are formed through a series of reactions initiated by 5-lipoxygenase. Due to the presence of 5-LO and LTC4 synthase, mast cells and eosinophils show a preferential production of LTC4 (279). Cysteinyl-leukotrienes, LTC4, LTD4 and LTE4, are important for causing bronchial constriction in asthma and play a role in CAD, rheumatoid arthritis, and allergic rhinitis. It has been noted that cysteinyl-leukotrienes are increased in ischemia–reperfusion injury in murine models and in patients (280, 281). On the other hand, LTB4 is secreted by mast cells, macrophages, and neutrophils. It functions as an autocrine chemoattractant of progenitor cells and immature cells (282). Atopic dermatitis patients express functional autoantibodies against IgE and/or FcεRI, and these antibodies have been shown to induce PGD_2_ and LTC4 by human cardiac mast cells to cause cardiac inflammation (283). Additionally, FcεRI-induced expression of COX-2, and presumably PGD2 biosynthesis but not LTC4, was shown to be, at least in part, controlled by miR-155 in human skin mast cells (284). Therefore, patients with autoimmune disease may be at increased risk of releasing vasoactive, proinflammatory mediators when cardiac mast cells get activated with autoantibodies against IgE or/and FcεR1 (283).

## 3. Mast cell receptors and the significance of toll-like receptors in inflammatory response

Mast cells express receptors for a variety of ligands (Figure 1). Some of the key receptors most relevant to this article are discussed here. Interaction of mast cell Fc receptors with anti-IgE or complement-bound pathogens is the most studied field of mast cell function through specific ligand binding (285–287). Additionally, mast cells utilize the TNF superfamily and TNF receptors (TNFR), CD30L-CD30, and pattern recognition receptors (PRRs) to engage in an array of interactions (288, 289).

PRRs enable mast cells to detect exogenous and endogenous signals and initiate immune surveillance. PRRs can detect, recognize, and neutralize pathogens by engaging the microbial pathogen-associated molecular patterns (PAMPs) and endogenous products containing damage-associated molecular patterns (DAMPs). PRRs include the C-lectin-type receptors (CLRs), retinoic acid-induced gene I-like receptors (RLRs), Nod-like receptors (NLRs) and Toll-like receptors (TLRs) (289, 290). Among these, certain TLR isoforms are relevant for the present discussion. Briefly, TLRs (isoforms 1–10 in humans, a total of 13 isoforms in murine species) are evolutionarily conserved, and their activation leads to an inflammatory response against microbial pathogens (291). TLRs are localized at the cell membrane or in the endosomes (TLR3, TLR7, and TLR9). Membrane-bound TLRs (TLR1, TLR2, TLR4, TLR5, TLR6, TLR10) contain a common intracytoplasmic domain that conveys signals by molecules that are shared by interleukin-1 (IL-1) receptor signaling to activate the NF-κB pathway and release pro-inflammatory cytokines (292).

Mast cells express most of the known TLR isoforms and among them TLR4 mediates responses to LPS (from the cell wall of Gram-negative bacteria) as well as heat shock protein (HSP60) (293–296). TLR2 recognizes several components of Gram-positive bacteria and mycobacteria, including peptidoglycan (PGN) and lipoteichoic acid (LTA). TLR2 activation results in release of cytokines like IL-4 but its activation by peptidoglycan (another TLR2 ligand) results in mast cell degranulation and histamine release (294, 297–300). Human endothelial cells constitutively express low levels of TLR2 and TLR4 and are activated by Gram-positive and Gram-negative bacterial components (48, 301, 302). The increased levels of TLR2 and TLR4 gene expression in the endothelium of human atherosclerotic lesions (303) and the reduced incidence of atherosclerosis in patients with TLR4 polymorphism (304), suggest a role for TLRs in vascular inflammation (305). The anecdotal observation that ‘acute coronary syndrome’ is common in patients following a viral or bacterial infection also supports a role for the TLR pathway in vascular inflammation and disease (306, 307).

## 4. Physiologically significant functions of mast cells

### 4.1. Overview of the physiological significance of mast cells

**Figure 2:**
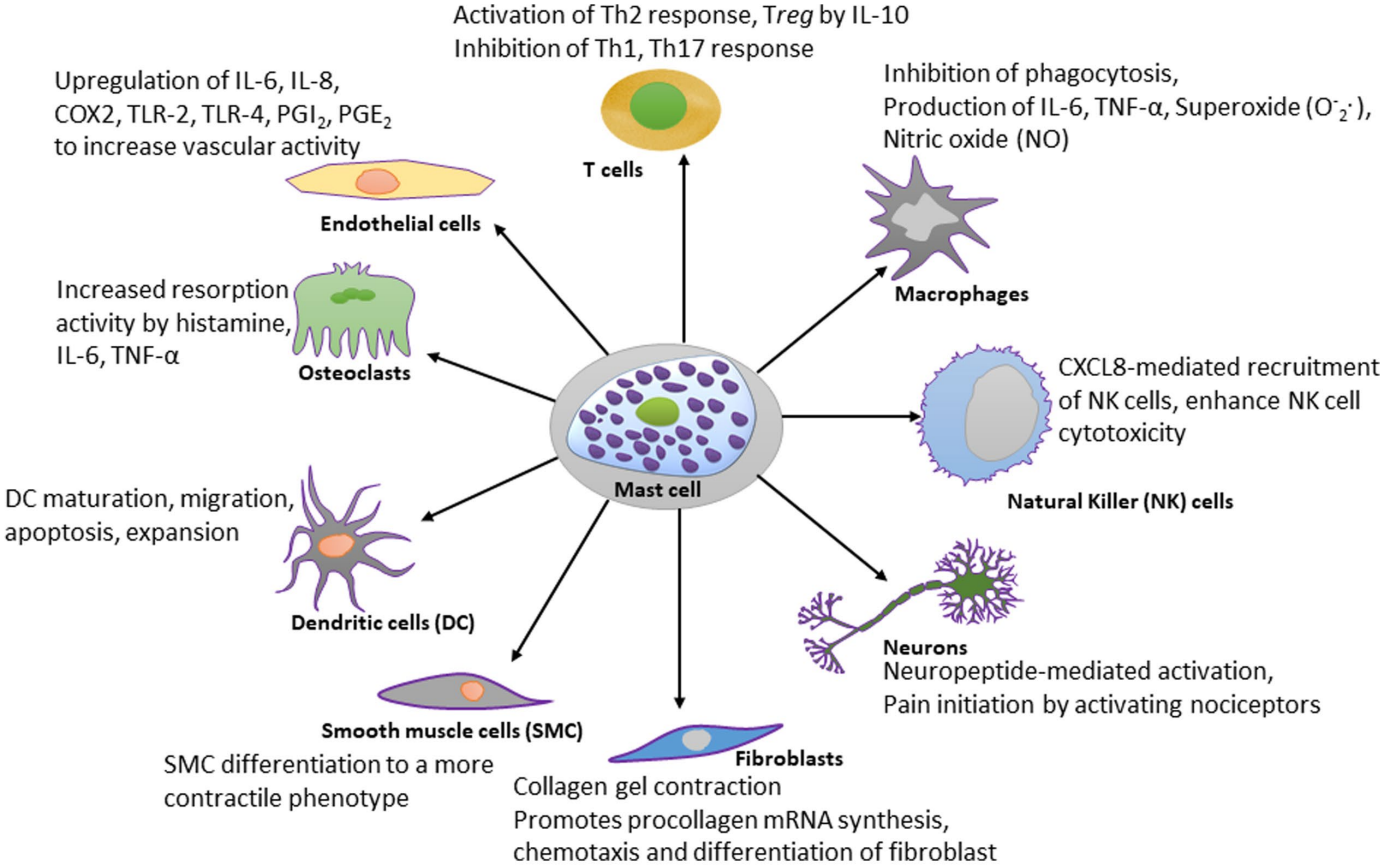
Schematic diagram showing the effect of mast cell-derived compounds on specific cell types. Mast cells have stimulatory or inhibitory actions on many types of cells. A few of those cell types are shown in this diagram. For the simplicity, the scheme shows only most studied effector molecules.

Mast cells are evolutionarily conserved cells dating back to thousands of years and their predecessor cells have been reported in non-vertebrate *Ciona intestinalis* (phylum chordata) prior to the evolution of adaptive immunity (308). Through their participation in both adaptive and innate immune systems by the virtue of their flexibility to change phenotypes with the microenvironment, mast cells participate in an array of physiological processes (Figure 2). Mast cells are present in almost all tissues, and are particularly notable in areas such as the mucosa in the respiratory, digestive, and urogenital systems, the dermis, blood vessels, lymph vessels, fibroblasts, and in the proximity of peripheral nerves, and this strategy allows them to function as sensors of changes in their local microenvironment (309). The localization of mast cells within the vessel wall highlights their involvement in the vasculature, specifically in vasodilatory functions and tissue-specific responses against circulating agents.

Mast cells play important roles in allergic and hypersensitivity reactions, vasodilatation, gastric acid production, angiogenesis, pathogen and parasite clearance, venom detoxification, mineral homeostasis, bone remodeling, wound healing, reproduction, and immune homeostasis, and each of these functions will be discussed.

Mast cells participate in immune homeostasis through multiple mechanisms of the innate and adaptive immune system (310–313). Although mast cells are not known as classic phagocytes, they are active participants in the elimination of bacteria through interactions with T and B cells and engaging ICAM-1, ICAM-3, CD43, CD80, CD86, and CD40L (73). Mast cells promote the development of Th2 cells and induce B cells to produce IgE through isotype switching (2).

The best recognized modulatory effects of mast cells are on dendritic cells, macrophages, T lymphocytes, natural killer (NK) cells, fibroblasts, neurons, endothelial cells, osteoclasts, and smooth muscle cells, and they are summarized in Figure 2. The significance of mast cells in neurodegenerative diseases is discussed separately.

### 4.2. Mast cells and infectious or toxic agents

#### 4.2.1. Mast cells and bacteria

The evidence supporting the role of mast cells in fighting bacterial infections is based on the finding that transfer of wild-type mast cells (TLR2^+/+^) reduced the pathogen load and myeloid cell recruitment in TLR2-deficient (TLR2^−/−^) mice infected with *Mycobacterium tuberculosis* (314). Furthermore, mast cell-deficient mice were found to be more susceptible to Group B *streptococcus* infection (315). Anti-bacterial response of mast cells is shown to be mediated by release of inflammatory molecules resulting in increased vascular permeability and fluid accumulation, and recruitment of immune cells (eosinophils, NK cells, and neutrophils) (41, 316, 317). Different bacterial genera have been shown to elicit distinct secretory responses from mast cells *in vitro* (318). Such specificity in response is due to receptors that are expressed in a tissue-specific microenvironment (discussed above). Mast cells also generate antibacterial molecules, such as cathelicidins, defensins, and psidins (2). Interaction of mast cells with bacterial toxins (LPS) is discussed separately in this article. Mast cells are important for maintaining the gut microbiome. In the gastrointestinal tract mast cells contribute significantly to the homeostasis of the commensal bacteria through modulation of IgA maturation and follicular helper T cells maturation (2).

#### 4.2.2. Mast cells and helminth infections

The controlled discharge of mast cell mediators through the activation of FcεRI by IgE is a vital component of the body’s defense against parasites, particularly helminths (319–322). In certain parasitic infections, mast cells are not significantly involved. Instead, the immune response relies on the binding of IgE to CD23 receptors found on eosinophils, platelets, macrophages, and dendritic cells. This leads to antigen presentation and the subsequent production of IgG antibodies (323, 324). In contrast, mast cell degranulation may be induced without involving IgE (322, 325). Despite its significance in some infections, mast cell involvement is neither crucial nor beneficial in all parasite-related responses. Mast cell degranulation can have harmful consequences, including tissue damage, and their activation is not always essential (326, 327). Nevertheless, parasitic helminths are sensed via tissue alarmins of the immune system that signal the presence of helminthic parasites leading to type 2 immune responses. Increased number of intestinal mast cells has been associated with Th2 immune response and certain cytokines (SCF, IL-3, IL-4, IL-9, IL-10, IL-18) (104). Notch 2 signaling pathway was found to mediate mobilization of mast cells to the area of parasite infection (328).

Mast cell degranulation following FcεRI activation by parasite-specific IgE and antigen is a strong response against certain parasites through several mediators. Mast cell proteases may directly act on parasites (329); glycosaminoglycans in mast cell granules may prevent adult worms from attaching to intestinal mucosa (330); cytokines/chemokines/mediators from mast cells modulate mobilization or function of other innate immune cells with various effects on parasites (331). Leukotriene LTB4 may recruit other immune cells to remove certain parasites (332). Mouse mast cell protease-6 (mMCP6) promotes eosinophil recruitment that may favor the parasite (333); mMCP1 released by mast cells may damage the intestinal barrier function thus promoting inflammation and pathological changes by degrading occludin and other tight junction proteins (334, 335).

Mast cells and basophils can release mediators that favor parasite expulsion through increasing vascular permeability (336–339). MC-mediated changes in the intestinal barrier function can promote the flow of fluid and blood-borne antibodies into the intestinal lumen creating an unfavorable environment for the parasites. Activation of IgE and/or IgG receptors creates cytotoxic milieu for parasite expulsion (340).

Overall, the response of mast cells to parasites depends on several factors including the type of parasite, presence of other parasites, genetic background of the host, status of intestinal mucosal cells and other surrounding cells, host microbiome, levels of parasite-specific vs. non-specific IgE, expression of high affinity (FcεRI) and low affinity (CD23) receptors for IgE, additional host defense cells including the granulating and non-granulating cells. Parasite-specific details, potential protective role of mast cells and treatment approaches are described in greater detail in more comprehensive reviews (24, 341).

#### 4.2.3. Protozoan infections (malaria)

Mast cells play a role in protozoan infections, but their actions can be both protective or harmful depending on factors such as parasite species, host genetics, and timing of infection. Both innate and adaptive immune systems are involved in this process (342). Histamine and mast cells promote disease severity (343, 344) and intestinal permeability in malaria (345). Mast cell activation during mosquito feeding leads to inflammatory response (granulocyte recruitment, lymph node hyperplasia) (346). Mast cells release TNF in response to IgE binding to FcεRI (adaptive immune response) as well as TLR4 binding to peroxiredoxin from the parasite (innate immune) (347, 348). Mast cell activation by mosquito saliva down regulates antigen-specific immune response through MIP2 and IL-10 and promotes disease through activation of tissue damaging CD8 + T cells (349). Malarial parasite antigen induces VEGF release from mast cells in cerebral malaria (350), increases co-infection (351). Mast cells activation by uric acid released from plasmodium-infected RBS leads to activation of the tissue damaging CD8+ T cells (342).

#### 4.2.4. Ixodid ticks

Mast cells along with basophils and IgE have been shown to contribute to host resistance to larval Ixodid ticks (352). These organisms can mediate the transmission of pathogens to the host. Such pathogens include the agents of Rocky Mountain spotted fever (Rickettsia rickettsia) (353), Q fever (*Coxiella burnetii*) (354), tularemia (*Francisella tularensis*) (355), granulocytic ehrlichiosis (*Ehrlichia ewingii*) (356), monocytotropic ehrlichiosis (*Ehrlichia chaffeensis*) (357).

#### 4.2.5. Fungal infections, venoms

Mast cells are also important players in the host immune response to fungi (358, 359). Based on the discovery that activated IgE can trigger the release of carboxypeptidase A, it has been established that mast cells play a crucial role in both innate and adaptive immune responses to a range of venoms. Carboxypeptidase A breaks down snake and bee venoms, and endogenous toxins. Detailed discussion of the significance of mast cells and IgE in toxic reactions to venoms as well as research on their role in host protection can be found in recent reviews (321).

### 4.3. Mast cells in the reproductive system

The role of mast cell mediators in reproductive biology including spermatogenesis and pregnancy is currently being investigated and reviewed recently (360, 361). In males, two phenotypes of mast cells (MC_TC_, tryptase-positive, chymase-positive, and MC_T_, tryptase-positive, chymase-negative) are present in the testis and epididymis. Additionally, histamine influences steroidogenesis in Leydig cells through H1 or H2R (362, 363). In females, mast cell proteases activate MMP2 and MMP9, resulting in matrix degradation during the menstrual cycle (364). Mast cell products are important for fertility, pregnancy (365), and abortion (366). Mast cells release histamine and α-chymase when activated by estradiol and progesterone (367) and contribute to uterine remodeling for embryo implantation. During childbirth, before the onset of labor, the number of mast cells in the cervix increases which contributes to cervical ripening (360, 368). In addition, increased number of mast cells (indicated by increased levels of TNF-α) is associated with abortion (366, 369).

### 4.4. Mast cells in the skin and wound healing

Mast cells localize close to endothelial cells of the vasculature, hair follicles and nerves (370, 371). Dermal mast cells also play a critical role in all stages of wound healing. Additionally, mediators released by mast cells show pro-inflammatory effects that promote acute inflammation. Mast cell mediators also stimulate scarring, epithelialization and angiogenesis (372). Further evidence shows that *in situ*-derived human skin mast cells can spontaneously secrete several angiogenesis-related factors leading to the speculation that mast cells could be involved in vasculogenesis as well as angiogenesis and human development (28).

Considerable evidence suggests that mast cells join other cells as modulators of acute skin wounds. The role of mast cells in wound healing has been summarized in recent reviews (373, 374). The process of wound repair in man and experimental models involves overlapping events. Studies using rodent models show that mediators released by mast cells appear to influence the changes during the entire process of wound healing. Studies show that, (i) mast cells number and degranulation along the wound area increase within minutes and reaches to maximum within 1–3 h after injury corresponding with clot (fibrin network) formation during the immediate local response/the hemostatic phase (375, 376). Mast cells release histamine and TNF that promote endothelial expression of adhesion molecules for leukocyte adhesion (253). Leukotrienes and cytokines from mast cells promote chemotaxis of neutrophils, basophils, and eosinophils. Mast cell secreted tryptase and cathepsin G modulate leukocyte adhesion and function and chymase may activate eosinophils to release chemokines (377, 378). After the initial rise, mast cell numbers return to the base line around 6 h. (ii) Vasodilation and plasma exudation with activation of phagocytes and granulocytes to clean the wound during the inflammatory phase lasts up to 2-3 days and continues into the next phase. Some authors suggest that attracting neutrophils during this phase is the main function of mast cells (379). (iii) A second increase in the number of mast cells is believed to contribute to the proliferative phase (24-72 h). Mast cells secrete growth factors and cytokines (TNF, TGF-β, proteases) that modulate proliferation of fibroblasts to induce their transformation into myofibroblasts and their function leading to wound closure (380), basal epithelial proliferation and movement to close/contract the wound during the proliferative phase (24-72 h). Mast cell secreted transforming growth factor-β (TGF-β), vascular endothelial growth factor (VEGF), chymase and tryptase stimulate angiogenesis (381–386) while heparin may block pro-angiogenic factors (387) (iv) remodeling phase, fibroblasts removed, deposition of collagen matrix and ECM resembling skin, scar formation and closure of the wound (2–3 weeks and longer) (388). During this phase, mast cell tryptase has been found to stimulate collagen synthesis by fibroblasts (389).

In the later phases of wound healing, histamine and serotonin act in a stimulating manner on epidermal keratinocytes, while TNF has an inhibitory effect on them (390). Additionally, mast cell proteases MCP4 and MCP6 play a role in minimizing scar formation in the central nervous system after trauma (321). Another study found accumulated mast cells and their products chymase, fibroblast growth factor 2 (FGF2), TGF-β1 and VEGF at the edge of scald wound in mice during the proliferative and remodeling phases at days 14 and 21. Chymase activity in the injured tissues was decreased in the acute phase, but recovered to a no-injury level at days 14 and 21 (386). Thus, mast cells influence wound healing by promoting acute inflammation, proliferation/re-epithelialization, and angiogenesis, as well as scar contraction and collagen cross-linking (372). The available data suggests that mast cells may play a role in chronic wound healing, but there is not enough conclusive evidence to make any definitive conclusions at this time (391, 392).

## 5. Abnormal mast cell activation or expansion

Although mast cells have been extensively studied for their pro-inflammatory effects, they are also known to cause anti-inflammatory effects (393, 394). The number and activation of mast cells are strictly regulated under normal physiological conditions and the increased number of mast cells and the overproduction and release of mediators by certain mast cell clones reflect pathological changes. Two well-defined conditions, namely mastocytosis and mast cell activation syndrome (MCAS), are associated with high numbers of mast cells and excessive release of pro-inflammatory mediators that may affect several organs. Mastocytosis is a rare disorder [1 in 10,000–20,000] that is characterized by clonal proliferation and accumulation of mast cells. Two types of mastocytosis have been described, namely cutaneous and systemic mastocytosis. Cutaneous mastocytosis indicates accumulation of mast cells in the skin without much infiltration elsewhere in the body and mainly affects children (395). In contrast, systemic mastocytosis is generally found among adults who present with large numbers of mast cells in multiple tissues besides the skin, namely the bone marrow, gastrointestinal tract, and lymph nodes (396, 397). Recent studies report increased incidence of cardiovascular diseases in patients with mastocytosis (398). Mast cell activation syndrome (MCAS) represents overproduction or over activation of mast cells due to a variety of etiologies. It leads to repeated episodes of a spectrum of multisystem dysfunctions and several aspects of MACS remain unclear (399). MCAS is currently being discussed in the context of inflammation and loss of alveolar function in some patients with SARS-CoV-2 infection (76, 399–401).

## 6. Mast cells and allergic diseases

Mast cells play a pivotal role in the pathogenesis of allergic diseases. Since mast cells have been recognized as a major contributor in initiating hypersensitivity reactions and the aftermath of the episodes leading progression of chronic diseases like asthma, atopic dermatitis and urticaria, considerable focus has been given to the field of mast cell biology research (402). Since it is far beyond the scope of this article to accommodate the vast volume of work done on the role mast cells in allergic diseases, we are providing only a very brief commentary in this section.

By virtue of their strategic locations in the skin, mucosa, gut and lungs, mast cells are in constant contact with the external environment and allergens (403–407). Mast cells are the effector cells responsible for the IgE-mediated allergic reactions in which allergens are recognized and presented by antigen processing cells to naïve T lymphocytes. T cells recognize the antigen as a foreign material and differentiate into Th2 lymphocytes. Mast cells also can process and present antigens via MHC-I and MHC-II complexes. Because of this characteristic feature, mast cells contribute significantly to the sensitization process and in directing the adaptive immune system toward Th2 pathway in response to antigens (408, 409). Allergies develop when components of the immune system, particularly mast cells, respond to antigens and release many prestored and newly synthesized mediators which in turn interact with a variety of cell types including endothelial cells, macrophages, epithelial cells, T and B lymphocytes, cardiomyocytes, parietal cells, and neurons. As mentioned earlier, mast cell-released mediators include histamine, serine proteases and other enzymes, proteoglycans (heparin or chondroitin sulphate) and as well as prostaglandins and leukotrienes which are rapidly synthesized from arachidonic acid by the enzymes, cyclooxygenase, and lipoxygenase, respectively (see mast cell released mediators above).

Earlier studies on the association of mast cells in allergic reactions were focused on the acute phase of these reactions. In this regard, FceR1 stimulation by a polyvalent allergen recognized by the receptor-bound IgE initiates immediate hypersensitivity reactions by releasing pre-formed and newly synthesized mediators from the sensitized mast cells. These mediators are responsible for allergic symptoms such as erythema, edema, increased vascular permeability, smooth muscle contraction and increased mucus secretion (410). The mast cell mediator such as histamine, PGD2 and LTC4 contributes to asthmatic symptoms, causing bronchoconstriction, mucus secretion and respiratory mucosal edema.

It should be emphasized that allergic reactions are complex and multiphasic comprising of both acute and chronic outcomes. In later episodes, proinflammatory mediators secreted by mast cells induce the recruitment of eosinophils, basophils, and T cells to the sites of inflammation (410–412). The late phase of the allergic reaction is followed by a chronic phase which is associated with persistent inflammation, tissue remodeling and fibrosis. These phases are prevalent in allergic asthma, rhinitis, and atopic dermatitis (413–415).

It is noteworthy that mast cells are also activated through IgE-independent mechanisms in the initial phases of an allergic response. Existing evidence suggests that serine proteases can directly activate mast cells (416, 417). In addition, a multitude of agonists including complement, neuropeptides, cytokines, stress hormones and radiocontrast chemicals can activate mast cells directly (418–421).

As described earlier, FceRI cross-linking with polyvalent antigens is the primary component initiating allergic responses. The uncontrolled and persistent mast cell activation can lead to life threatening conditions such as anaphylaxis and chronic inflammatory diseases like asthma. Therefore, many recent therapeutic strategies are aimed at targeting this pathway to inhibit mast cell degranulation and mediator release (422).

## 7. Mast cells and atherosclerosis

The pathogenesis of atherosclerosis involves endothelial cell damage, persistent inflammatory response, increased expression of adhesion molecules and accumulation of a variety of cell types (423–426). There is ample evidence in the literature to demonstrate the role of mast cells in vascular inflammation and atherosclerosis progression (2, 57, 87, 120, 220, 221, 231, 236, 237, 427, 428). Pro-inflammatory and anti-inflammatory substances released by mast cells (175–178) are important modulators of vascular homeostasis particularly in relation to atherosclerosis and cardiovascular diseases (115, 116, 118, 120, 124, 134, 136–139, 429–433). Pro-inflammatory activation of vascular endothelium by mast cell mediators, cytokines such as TNF and IL1-β, bacterial cell wall components, viruses, and thrombin can induce local thrombosis, endothelial production of cytokines, loss of vessel barrier function, and enhanced leukocyte adhesion. Such chronic or subclinical inflammatory activation of the endothelium is a part of host defense mechanisms, and its dysfunctional response leads to early events in the onset and progression of atherosclerosis (434, 435).

### 7.1. Mast cells as mediators of inflammation during the initial stages of atherosclerosis

Mast cells play a critical role in the complex process of immune response during acute and chronic inflammatory states and they are implicated in the progression of atherosclerosis (59, 87, 217, 231, 236, 237, 423–426, 436). The adventitia of coronary arteries of patients with atherosclerotic plaques contain increased number of mast cells (57, 120, 231, 427, 437). Histological studies have demonstrated several hundred-fold increase in the number of activated mast cells in the atherosclerotic plaque rupture regions of coronary arteries. Mast cells in degranulated state were considered to have already undergone activation (438). Besides the adventitial layer, epicardial adipose tissue around the coronary artery also contains increased number of mast cells in CAD patients compared to controls (439). Increased number of mast cells were reported in the adventitia of the thrombosed veins, in vicinity of the vasa vasorum (440). Mast cells are considered, at least partially, to contribute to thrombosis through histamine release and by stimulating endothelial activation (441).

Mast cell granules have been identified within endothelial cells *in vivo* (442) and are known to cause proliferation of human microvascular endothelial cells (443). Furthermore, mast cell granule remnants have been shown to bind to low-density lipoproteins (LDL) and enhance their uptake by macrophages leading to the development of foam cells (236, 237).

### 7.2. A dual role for mast cells in infection-induced inflammation

A potential role for infection in the development of atherosclerosis has been considered for several decades. The first experimental evidence for infection-induced atherosclerosis was demonstrated in chickens using the herpes virus model (444). Later, many infectious agents were found to be associated with atherosclerosis. Interest in this topic has re-emerged because of several observations (445–451).

Vascular endothelial cells are critical targets for microbial pathogens and their activation by LPS results in the production of various inflammatory cytokines, chemokines, and cell adhesion molecules. The role of mast cells in these processes was demonstrated by a synergistic enhancement of LPS/LTA-induced production of IL-6 and IL-8 production by endothelial cells (48, 239). These amplifying effects have been shown to be due to histamine-induced overexpression of active TLR2 and TLR4 on endothelial cells (48). Furthermore, in a follow-up study, we demonstrated that LPS induces the expression of H1 receptor in endothelial cells (50). Collectively, these bidirectional effects of histamine and bacterial cell components lead to amplified inflammatory responses in the vascular endothelium via upregulation of the expression and TLR2, TLR4 and H1 receptors. Therefore, persistent infections together with mast cell degranulation provide ideal environments for the progression of atherosclerosis.

It is well-recognized that the immune response to bacterial products may recruit and sensitize inflammatory cells including mast cells, to potentiate localized inflammation. Microorganisms such as cytomegalovirus, *Chlamydia pneumoniae* and *Helicobactor pylori* are found in atherosclerotic lesions (426, 452, 453). The relationship between infection and inflammation to atherosclerosis has been further strengthened by the recognition of *C. pneumoniae* in patients with the disease (454–456). *C. pneumoniae,* which localizes in human atheroma, may contribute to inflammation during atherogenesis by activating endothelial cells, smooth muscle cells and macrophages (457–460). These findings are consistent with the risk of myocardial infarction and stroke observed in patients with systemic and respiratory tract infections (461). Therefore, it can be postulated that a cooperative action of bacterial products and mast cell mediators may amplify endothelial cell activation and progression of atherosclerosis.

### 7.3. Mast cells and inflammation linked to changes in microvessel integrity and, plaque growth and disruption

The development of atherosclerosis involves highly coordinated involvements of macrophages, mast cells and subsets of T cells (462–465). Mast cells strategically reside near microvessels, but very few are localized in the vascular intima. In contrast, the adventitia is densely populated with mast cells (466, 467). In conditions of persistent vascular inflammation and onset of atherosclerosis progression the mast cell population increases in the adventitia. Whether the increased mast cell population in the plaque region promotes atherogenesis or does it enhance plaque stability or both, is an open question. However, the available reports suggest that mast cells promote vascularization in atherosclerotic plaques by providing angiogenic factors such as VEGF-A and β-FGF as well as other proangiogenic mediators like heparin, tryptase and chymase (468, 469). Because neovascularization and vasodilation can improve oxygen supply to the affected hypoxic areas of the plaque, this process can stabilize the atherosclerotic plaque (470). It should be emphasized that mast cell proteases can degrade basement membranes of microvessels in the plaque causing intraplaque hemorrhage and plaque rupture (463).

Mast cells migrate into the plaque by the influence of chemokine CCL-1 present in the plaque, and CCR-2 expressed on the mast cell surface (217, 471). An increased number of tissue mast cells was suggested to be associated with thrombus formation mast cells (437). Mast cells in atherosclerotic plaques may also contribute to atherothrombosis through the formation of DNA extracellular traps (267, 472). Thus, the effects of activated mast cells in the neovascularized regions of atherosclerotic plaques appear to be both protective and harmful. This might be due to the unique characteristics of the mast cell to differentiate into different phenotypes based on the microenvironment. In this regard, mast cell mediators have been shown to play protective roles via immune homeostasis in a mouse model of systemic vasculitis (473).

Upon degranulation, mast cells release histamine and matrix-degrading proteases, which can cause microvessel leakage and rupture, leading to intraplaque hemorrhage. Mast cell activation during the progression of atherosclerosis has been shown to increase plaque size in the brachiocephalic artery of apoE-deficient mice (221). This response was prevented by administering cromolyn, a mast cell stabilizing agent. Role of mast cells in the progression of atherosclerosis has been further confirmed by findings that mast cell deficiency attenuated the development of atherosclerotic plaque in both LDL receptor-deficient *LDLr^−/−^* /*Kit^W-sh/W−/sh^* mouse model (87) and apoE-deficient apoE*^−/−^/Kit^W-sh/W−/sh^* mouse model (436).

The work summarized in this section supports a major role of mast cells in vascular inflammation, progression of atherosclerosis and plaque rupture. On the other hand, mast cells can exert protective effects in other vascular diseases such as systemic vasculitis. We believe that systemic vascular changes influence other organs. Tissue-specific resident mast cells participate in response to systemic vascular changes through sensing inflammation and then participate in the response mounted distally.

## 8. Mast cells mediate vascular inflammation caused by microbial infection and lifestyle choices such as cigarette smoking

Cigarette smoke extract-treated mast cells induce macrophage infiltration and M2 polarization (474). Recent studies have shown that cigarette smoke affects mast cell development and their response to activation in a TLR4-independent manner. Cigarette smoke attenuates the granularity and expression of surface c-kit and FcεRI in maturing mast cells resulting in decreased degranulation and release of Th1 and Th2 cytokines upon stimulation (475). Cigarette smoke induces the expression of chemokines and serine protease member S31 in mast cells (474).

Cigarette smoke increases susceptibility to bacterial infection and infection-related mortality among smokers (476–478) and mast cells respond to both cigarette smoke and microbial toxins. Since both endothelial cells and mast cells express TLRs that are activated by microbial toxins (48, 50, 479), pro-inflammatory constituents of cigarette smoke create conditions favorable for endothelial activation and mast cell degranulation. As mentioned previously, histamine activates endothelial cells via H1R (48, 436). These observations led us to propose that cigarette smoke amplifies activation of endothelial cells and inflammation through H1R-TLR2/4-COX2 axis (480). Thus, our results indicate a synergistic effect of cigarette smoke and the bacterial toxin LPS on endothelial cells in the presence of histamine.

## 9. Mast cells and viral infection with special reference to severe acute respiratory syndrome coronavirus-2 (SARS-CoV-2)

Research on the role of mast cells in viral infections is relatively new compared to the extensive work on bacterial infections and bacterial toxins. Viral infections are associated with mast cell-mediated aberrant inflammatory response, vascular leakage, and fibrosis (479, 481). Mast cells participate in host defense against viruses recruiting CD8+ T cells, which produce IFN-α and IFN-β. Dendritic cells function as antigen presenting cells and mast cells support dendritic cells for T-cell activation in adaptive immunity (321). Thus, while some of these actions are directly mediated by them, mast cells may also prompt or tune other innate, adaptive, or structural cells to engage in physiological actions. Influenza A virus infection was shown to activate mast cells leading to the release of histamine, proteases, leukotrienes, inflammatory cytokines, and antiviral chemokines (482). HIV-1 viral infection-mediated immunosuppression has been found to be linked to mast cell-released histamine (483).

The recent pandemic caused by SARS-CoV-2 appears to primarily affect the dense capillary network in the lungs causing high morbidity and mortality. Published data suggest a role for mast cells in the pulmonary complications associated with SARS-CoV-2 infection. Hyperactivation of proinflammatory cytokines [‘the cytokine storm’] is detected in nearly 20% patients with COVID-19 who experience a severe course of the infection for reasons [s] not well understood. Similar cytokine storms also characterize a multisystem disorder associated with idiopathic MCAS affecting approximately 17% population (301, 399). In line with that, higher numbers of mast cells were detected in the lungs of patients who died of COVID-19 infection (484).

SARS-CoV-2-triggered MC degranulation leading to alveolar epithelial inflammation and lung injury has been reported in ACE-1 humanized mice and rhesus macaques (485). Several molecules expressed/released by mast cells may contribute to the interaction between mast cells and SARS-CoV-2. Mast cells express the surface angiotensin converting enzyme 2 (ACE2) receptor of the renin-angiotensin system that is considered to be a candidate receptor for SARS-CoV-2 binding to cells (486, 487). A role for histamine released by mast cells in lung inflammation associated with SARS-CoV-2 infection was proposed almost immediately after the outbreak of the COVID-19 pandemic (75). Later, a report showed that histamine signaling through H2 receptor was essential for SARS-CoV-2 spike protein-mediated ACE2 internalization in endothelial cells (488). Mast cell-derived serine proteases, e.g., transmembrane serine protease 2 (TMPRSS2) facilitate the priming of the corona spike protein, and tryptase also has a role in SARS-CoV-2 infection (489, 490). Tryptase levels have been found to be associated with the severity of COVID-19 (491, 492). Tryptase secretion from mast cells activated via complement and IgE-dependent pathways is much higher compared to their direct activation via Mas-related G Protein coupled receptor X2 (MRGPRX2) (493). Tryptase hast been known to activate PAR2 on fibroblasts and induce collagen synthesis, fibroblast proliferation, and migration, airway remodeling/lung and development of fibrosis (494, 495). Although a direct link between mast cell proteases and matrix degrading metalloproteases (MMP) in COVID-19 remains to be established, elevated levels of plasma MMP2 and MMP9 have been shown to be associated with the severity and mortality of patients with COVID-19 (496). MMP2 and MMP9 were found to facilitate viral entry into the cell and were postulated to have a role in cytotoxicity of the virus and outcome of the disease (497). Increased MMPs (MMP8, MMP9, and MMP14) and matrix degradation in the lungs of mice infected with SARS-CoV-2 have been reported (497, 498). In support of activation of MMPs by tryptase, mast cell tryptase has been shown to activate lung MMP1 from pro-MMP1 in asthma and increase disease severity (499).

Aside from eicosanoids and other mast cell-released molecules, IL-1 is uniquely positioned to cause tissue damage due to its broad spectrum of biological effects and role in both innate and acquired immunity. Additionally, IL-1 induces multiple cytokines/chemokines in both macrophages and mast cells. Release of IL-1 following SARS-CoV-2 infection increases the levels of TNF, IL-6, and other cytokines. IL-1 also induces upregulation of nitric oxide, prostaglandins, and TXA2. Thus, IL-1 may be responsible for the ‘cytokine storm’ and inflammation, lung disease and death. Anti-IL-1 agents may be a valuable new line of treatment for SARS-CoV-2. In this regard, anti-inflammatory effects of IL-37, IL-1Rα are being studied to neutralize the effects and levels of IL-1 (75, 76).

**Figure 3:**
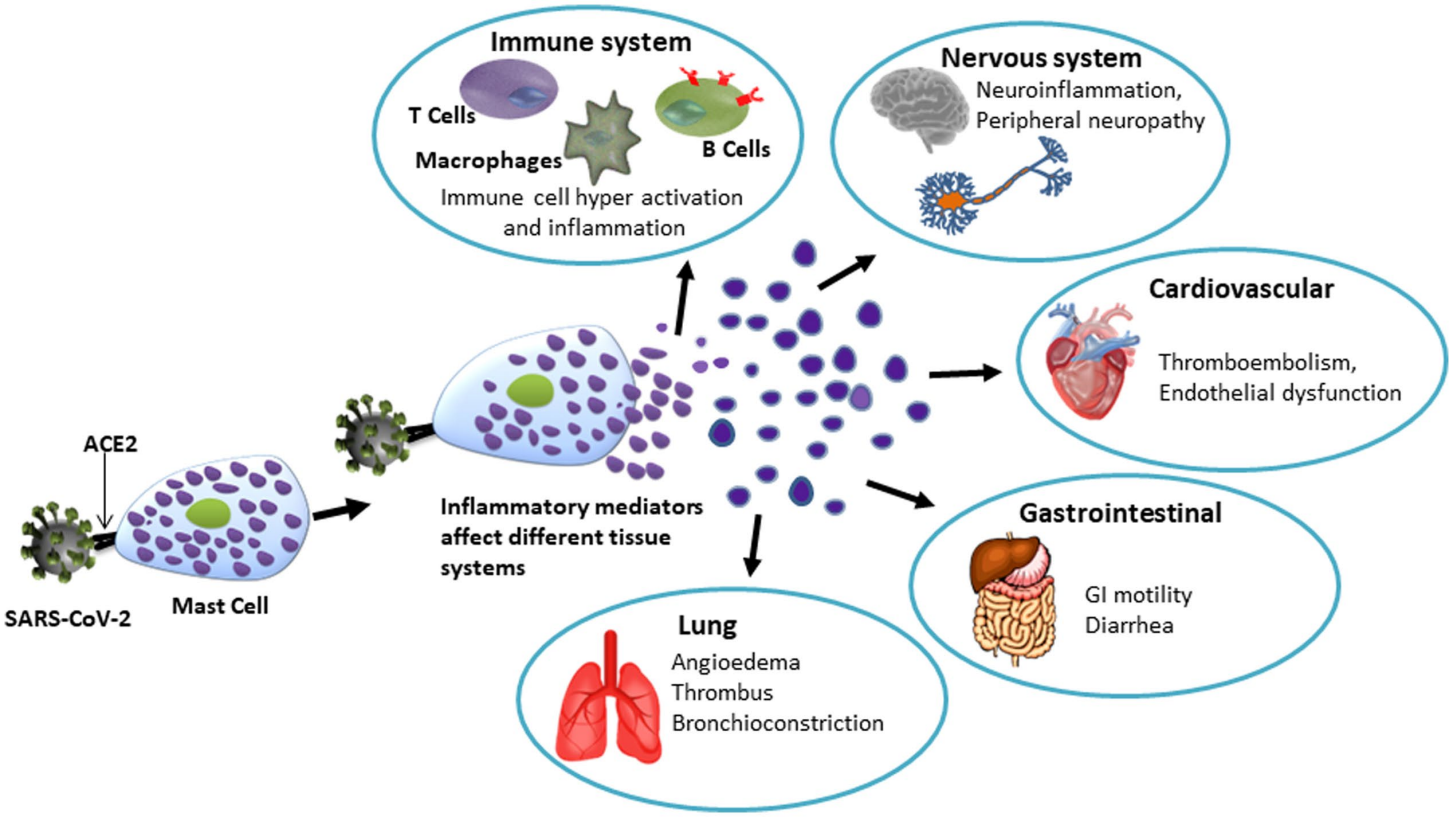
Schematic representation of the potential role of mast cells in SARS-CoV-12- induced tissue/organ damage. Spike protein of SARS-CoV-2 binds to ACE2 receptor on mast cells to cause degranulation. The hyperactive mast cells-derived granules contain various bioactive molecules that affect organ systems as mentioned. Notably, mast cell activation is now considered to be a key mechanism involved in various organ-related symptoms indicating long COVID.

Earlier studies have shown that, in addition to activation of monocytes/macrophages, dendritic cells, T cells, mast cells, and neutrophils, and the induction of cytokine storm in the lung, COVID-19 also affects other cells including neurons, glial cells, and endothelial cells thus causing neuroinflammation and psychological stress (500). A recent report showed the presence of CD117+ cells and IL-4-expressing cells in perivascular and alveolar septa that provide the interface between capillaries and the environment for gas exchange in the lungs in post-mortem biopsy samples of COVID-19 patients. The number of mast cells found in these biopsies were higher than previously reported in samples collected from patients with pandemic H1N1-induced pneumonia. Resulting upregulations of chemokine/cytokines cause alveolar injury and immune-thrombosis (484). However, it remains to be proven that endothelial dysfunction due to hyperactive mast cells results in interstitial edema in the alveolar septa triggering diffusion of pro-coagulative plasma factors leading to fibrin-dependent generation of the hyaline membrane. It is also noteworthy, that abnormally high mast cell activation has been observed in ‘long COVID/post-COVID syndrome’ where some patients experience lasting effects of SARS-CoV-2 infection in different organs (501, 502). A perspective on the involvement of mast cells in SARS-CoV-2-mediated organ damage is illustrated in Figure 3.

## 10. Mast cells as effectors for integrating systemic inflammation, neuroinflammation, and neurodegenerative diseases

In the brain, mast cells are mostly localized in the immediate vicinity of blood vessels and cerebrospinal fluid (CSF)-containing spaces (503) notable in the hippocampus and thalamic hypothalamic region, 3^rd^ ventricle, choroid plexus and innermost meninges (leptomeninges) (504–508). The number of mast cells in the human brain is low compared to the rodent brain and these cells are found mostly along meninges and perivascular regions (509–511). Studies using rat and human brain tissues show that majority of mast cells (>95%) localize on the abluminal side of blood vessel (brain side) during development (507, 512). However, peripherally injected mast cells were also found to enter the basal lamina (matrix) of blood vessels and localize close to astrocyte (astroglial) processes and microglia (506, 513, 514). Such localization allows mast cells to perform their role as transducers of signals and as first line defenders (515). Activation and proliferation of mast cells due to injury-induced derangement of blood–brain barrier in traumatic brain injury (TBI) suggests that mast cells may be potential targets for therapeutic approaches (516).

### 10.1. Mast cell-microglial interactions

Interactions between mast cells and microglia are a subject of growing research interest because unique structural and functional features of microglia allow mast cells to influence both physiological functions as well as pathological changes (511, 517). Structural and molecular features enable reciprocal interaction between mast cells and microglia (518) discussed under the following three categories (a-c).

#### 10.1.1. Reciprocal interaction between mast cells and microglia

(a) P2 channel/receptor activation by ATP increases IL-33 release in activated microglia (by PAMPs via TLRs). In turn, IL-33 binding to mast cells induces IL-6, IL-13 and MCP-1 secretion that modulates microglial function (519–521). (b) activation of TLR2/4 induces phagocytosis and production of IL-6 and CCL5 in microglia that induce TLR2/4 expression in mast cells. In turn, activation of TLR2/4 in mast cells upregulates cytokine/chemokine formation. CCL5/RANTES activates microglia and promotes immune cell movement to the location of injury (522, 523). (c) Mast cell tryptase activates PAR2 in microglia causing increased synthesis and release of BDNP, increased release of pro-inflammatory molecules (IL-6 and TNF) by upregulating MAPK-NF-kB. In turn, IL-6 and TNF from microglia upregulate PAR2 on mast cells (524).

#### 10.1.2. Mast cells and microglia as dual targets of inflammatory molecules/conditions

(a) Binding of chemokine (CXCL12) to its receptor (CXCR4) induces microglial activation and mobility under ischemia/hypoxia. CXCL12 is also a chemotaxin for mast cells (525). (b) Co-culture of mast cells with glial cells caused increased synthesis of CCL2 and IL-33 by mast cells and acceleration of Aβ oligomerization by microglia. CCL2 (monocyte chemoattractant protein-1 or MCP-1), and its receptor CCR2 are expressed on monocytes. Several recent studies suggest increased CCL2 in Alzheimer’s disease (AD) (516). Elucidation of the significance of CCL2-CCR2 axis in AD requires further studies. (c) Neuroinflammation induces release of complement protein C5a and upregulation of C5aR in microglia. Similarly, activation of mast cells is associated with C5aR upregulation attracting C5a peptides. Both cell types show crosstalk between C5a and TLR4 (526).

#### 10.1.3. Activated mast cells modulate microglial function

(i) Experimental release of granules from mast cells upregulates histamine receptors, MAPK, AKT proteins and cytokine production in hypothalamic microglia (511, 527). (ii) Mast cell-released histamine elicits a pro-inflammatory response in quiescent microglia through H1 receptor and an anti-inflammatory response in activated microglia through the H4 receptor (528, 529).

### 10.2. Significance of mast cell-neural cell interactions in CNS pathophysiology

Neuroinflammation drives the processes leading to acute as well as progressive neurodegeneration. A growing number of studies suggest that neuroinflammation is not isolated from peripheral conditions and that mast cells may play a significant role through their interaction with neurons, astrocytes, and glia, particularly with microglia (see above).

Increased numbers and activation of brain mast cells are associated with various neuropathological changes starting with increased blood brain permeability and leading to neurodegenerative diseases. In addition, their activation in response to physical injury and inflammatory conditions, the role of mast cells in the pathophysiology of neuropsychiatric conditions is also a subject of growing interest (530). Due to their ability to respond in a very short time, mast cells have been considered as potential ‘first responders’ (531). The following outlines the significance of mast cells in selected neuropathological conditions including TBI, stroke, Alzheimer’s disease, and certain neuropsychological disorders.

#### 10.2.1. Traumatic brain injury (TBI), hypoxia, ischemia, and intracranial hemorrhage

Neuroinflammation is associated with TBI that may lead to neurodegenerative changes often observed in AD-like dementias. Tissue injury due to hypoxia, ischemia (stroke) and intracranial hemorrhage also results in mast cell accumulation and degranulation with common features. Persistent inflammation due to moderate and severe TBI causes progressive tissue damage and has been linked to a greater susceptibility to AD-like dementia (532–534). Long term effects of TBI in humans and mouse models include cognitive deficit, deposition of amyloid precursor protein (APP), amyloid-beta peptide and tau protein intracellular neurofibrillary tangles (535, 536).

Immediate increases in the number and degranulation of mast cells in the injured brain area have been demonstrated in experimental models. Mast cell degranulation were shown to start immediately after TBI in a mouse model (537). Mast cell numbers increase rapidly along with the release of mediators including histamine, heparin, leukotrienes and tryptase and changes in histamine receptors (decreased H1, H3) and superoxide dismutase (SOD) in the brain (thalamic region) after TBI in rats (538). Persistent high number and degranulation of mast cells in the dura in a mouse model was correlated with post-traumatic headache in TBI patients (539).

Ischemia/hypoxia involve increased numbers and activation of mast cells indicated by increased brain histamine levels and increased cytokines (540, 541). These changes result in increased blood brain permeability (542, 543), interaction with microglia and influx of peripheral leukocyte leading to prolonged inflammation and consequent demyelination (508, 544, 545). Changes in the blood brain barrier characteristics are invariably linked to its constituents, namely endothelial cells and astrocytes (546). Due to shared tissue changes and injury, mast cells are considered as targets for treating TBI, hypoxia-ischemia, ischemic stroke, and intracranial hemorrhage (547).

#### 10.2.2. Alzheimer’s disease (AD)

Inflammation in Alzheimer’s disease has been a subject of ongoing investigation and the role of mast cells in neuroinflammatory processes of AD continues to be studied (548–550). Higher numbers of tryptase positive mast cells and metallothionein positive astrocytes have been observed near amyloid plaques in patients with AD (551). Likewise, microglial clusters around plaques have been reported in human specimens as well as in mouse models (552). Glial clusters secrete proteins and chemoattractant for mast cells (552). However, the idea of mast cells being ‘first responders’ suggests that these cells reach the plaque site and activate microglia and other cells (553). In either case, as discussed above, a reciprocal interaction between the two cell types is clear. In a rodent model (APPswe/PS1dE9 mice) of AD, increased numbers of mast cells were reported in the hippocampus and cerebral cortex even before amyloid plaques were observed. This led the authors to hypothesize that mast cells act as early sensors of amyloid peptide and facilitate mobilization of other cells to the site of inflammation promoting the onset and progression of AD (554). Cytokines including TNF, IL-1β, and IL-6 released by mast cells can contribute to toxic neuroinflammation as well as to cyto-protective processes determined by their levels and duration of expression. These cytokines may affect tight junction proteins resulting in altered blood brain barrier permeability and mobilization of circulating pro-inflammatory cells and molecules. Attenuation of cognitive decline by tyrosine kinase inhibitor masitinib that targets mast cell through the receptor c-kit and Lyn tyrosine kinase was concluded to indicate a role of mast cell-released mediators in progression of AD (555, 556).

#### 10.2.3. Anxiety, depression, and behavioral disorders

Cross-sectional human studies showed association of food allergies with anxiety (557) attention deficit and hyperactivity disorder (558) and anorexia nervosa (559, 560). Analysis of data on food allergies in children showed association of these conditions with changes in emotions and behavior. Using a mouse model of food allergy authors showed increased cortisone levels, changes in brain areas associated with emotional and affective behavior (anxiety and stress responses) that were partially mediated by C-sensitive afferent inputs and mast cells (561). Increased incidence of headache (migraine) anxiety, depression, and cognitive changes in human subjects have been reported to associate with mastocytosis (562, 563).

## 11. Concluding comments and perspectives

Mast cells are recognized for their role in maintaining immune homeostasis in health as well as in the pathobiology of many diseases. They are strategically residing throughout the body in almost all organs. Most of the published work has focused on their roles in skin, connective tissues, blood vessels, mucosa, lungs, and heart. Mast cells function as a cellular interface between the external and internal environments and initiate and coordinate innate and adaptive immune responses by interacting with a variety of cell types. The ability of the mast cell to act both as a sensor and responder while maintaining flexible mobility and adaptability enables this “master cell” to play a leading role in the complex process of immune homeostasis. In this article we highlighted selected functions of the mast cell to describe how these functional alterations affect the pathobiology of diseases.

Our work has been focused on understanding the mechanisms of mast-cell mediated immune alterations in cardiovascular diseases. In this regard, the synergy between mast cells and endothelial cells and the suppressive effects of mast cells on macrophage functions are intriguing findings. We have coined a novel term “H1R-TLR-COX2 axis” to describe these interactions. Chronic or sub-clinical infections and lifestyle choices such as cigarette smoking add additional dimensions to innate immune upregulation in cardiovascular diseases.

We also wish to bring attention to the role that mast cells play in angiogenesis which may have implications for tissue growth and wound healing. A brief review of their role in tissue repair processes points to future success of the ongoing efforts in exploring the potential using mast cells as target for developing novel treatments for diabetic wounds. On the flip side, inhibition of the pro-angiogenic role of mast cells is being explored for suppressing tumor growth. In this regard, we believe there will be much to gain by understanding the selective secretion of mediators by mast cells.

We hope that discussion on the role of mast cells as ‘first responders’ in neuroinflammation will stimulate greater interest in the field of pathobiology of neurological diseases. The significant influence of mast cells on both sides of the blood–brain barrier adds a new dimension to the field of developing therapeutic strategies for systemic vascular biology dysfunctions in cardiovascular and neurovascular diseases.

We believe that mast cells stand in the forefront of candidates for future work for exploring the impact of immunomodulatory dysfunctions in health and disease. It is nature’s rule that there are no “Good Guys” or “Bad Guys,” but it is the environment that influences the switch. Although a great amount of information has been gathered regarding the physiological role of mast cells, much remains to be learned about their participation as the ‘good guys’. Perhaps a better understanding at the epigenomic level will enable a greater utilization of their anti-inflammatory functions at the interface of tissues and the vasculature.

## Author contributions

KND, RaS, VVR, and MS have collaborated on several projects and proposed the premise for this article. MS, VVR, and KND revised the manuscript and figures. MS, VVR, and KND completed the final version. All authors contributed specifically to individual area of specialty, participated in revising the manuscript, contributed to the article, and approved the submitted version.

## Funding

Most of our research work cited in this article was supported by grants from NIH (R01-HL070101 and 3R01-HL070101-04S1), American Heart Association, Joseph and Elizabeth Carey Arthritis Fund, Lied Foundation, and the Audrey E. Smith Medical Research Fund from the University of Kansas Endowment Association (KD). MS received support, in part, from the Midwest Veterans’ Biomedical Research Foundation (MVBRF) and the Kansas City VA Medical Center, Kansas City, MO.

## Conflict of interest

The authors declare that the research was conducted in the absence of any commercial or financial relationships that could be construed as a potential conflict of interest.

## Publisher’s note

All claims expressed in this article are solely those of the authors and do not necessarily represent those of their affiliated organizations, or those of the publisher, the editors and the reviewers. Any product that may be evaluated in this article, or claim that may be made by its manufacturer, is not guaranteed or endorsed by the publisher.
